# 3D‐Printed Hydrogel‐Based Flexible Electrochromic Device for Wearable Displays

**DOI:** 10.1002/advs.202404679

**Published:** 2024-08-09

**Authors:** Xiaoyu Luo, Rongtai Wan, Zhaoxian Zhang, Manting Song, Lixia Yan, Jingkun Xu, Hanjun Yang, Baoyang Lu

**Affiliations:** ^1^ Jiangxi Province Key Laboratory of Flexible Electronics Flexible Electronics Innovation Institute Jiangxi Science and Technology Normal University Nanchang Jiangxi 330013 P. R. China; ^2^ School of Pharmacy Jiangxi Science and Technology Normal University Nanchang Jiangxi 330013 P. R. China; ^3^ School of Chemistry and Materials Science East China University of Technology Nanchang Jiangxi 330013 P. R. China; ^4^ School of Chemistry and Chemical Engineering Jiangxi Science and Technology Normal University Nanchang Jiangxi 330013 P. R. China

**Keywords:** 3D Printing, camouflage, flexible electrochromic device, hydrogels, wearable displays

## Abstract

Flexible electrochromic devices (FECDs) are widely explored for diverse applications including wearable electronics, camouflage, and smart windows. However, the manufacturing process of patterned FECDs remains complex, costly, and non‐customizable. To address this challenge, a strategy is proposed to prepare integrated FECDs via multi‐material direct writing 3D printing. By designing novel viologen/polyvinyl alcohol (PVA) hydrogel inks and systematically evaluating the printability of various inks, seamless interface integration can be achieved, enabling streamlined manufacturing of patterned FECDs with continuous production capabilities. The resultant 3D‐printed FECDs exhibit excellent electrochromic and mechanical properties, including high optical contrast (up to 54% at 360 nm), nice cycling stability (less than 5% electroactivity reduction after 10 000 s), and mechanical stability (less than 19% optimal contrast decrease after 5000 cycles of bending). The potential applications of these 3D‐printed hydrogel‐based FECDs are further demonstrated in wearable electronics, camouflage, and smart windows.

## Graphical abstract

The manufacturing process of patterned flexible electrochromic devices (FECDs) complex and non‐customizable. To address this challenge, a strategy is proposed to prepare integrated FECDs via multi‐material direct writing 3D printing. The FECDs exhibit impressive electrochromic and mechanical properties, including high optical contrast and excellent cycling stability, and is shown to have great potential in wearable displays, camouflage, and smart windows.

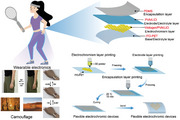

## 1 Introduction

Flexible electrochromic devices (FECDs) have attracted increasing attention due to their broad prospects in various fields such as wearable electronics, smart windows, flexible displays, and energy conservation.^[^ ^1^, ^2^, ^3^, ^4^, ^5^ ^]^ A variety of patterned FECDs processes have been explored in this field, including inkjet printing,^[^ ^6^ ^]^ electrochemical polymerization,^[^ ^7^ ^]^ screen printing,^[^ ^8^ ^]^ and electrospinning.^[^ ^9^ ^]^ However, traditional FECDs are typically designed as five‐layer structures, consisting of a base layer, an electrode layer, an electrochromic layer, an electrolyte layer, and an encapsulation layer. Their integrated manufacturing processes often involve complex procedures such as multi‐step processes in clean rooms, including alignment, masking, etching, and post‐assembly. This process inevitably leads to production challenges such as high equipment costs, complex process flows, and insufficient production capacity.

3D printing technology has emerged as a research hotspot due to its programmability, high efficiency, and low cost, among other advantages.^[^ ^10^, ^11^, ^12^, ^13^, ^14^, ^15^ ^]^ It has significant potential application prospects in various fields such as biomedicine, chemistry, aerospace, and automotive industries.^[^ ^16^, ^17^, ^18^, ^19^, ^20^ ^]^ Notably, 3D printing has the potential to enable customized, seamlessly integrated manufacturing of FECDs. However, several challenges still need to be addressed to achieve integrated FECDs through 3D printing, including the difficulty in selecting electrochromic inks and the complex process of 3D printing multi‐layer flexible electrochromic devices. Multi‐layer FECDs printing requires the coordination of multi‐material inks with different viscoelasticities to achieve the printing of different device layers, shape fidelity control, and seamless interface integration.

Among numerous electrochromic materials, hydrogels have exhibited great advantages in the development of FECDs due to their soft, highly bendable, stretchable, and self‐healing properties.^[^ ^21^, ^22^, ^23^, ^24^, ^25^ ^]^ Polyvinyl alcohol (PVA) is widely recognized as an ideal matrix for preparing various functional hydrogels, due to its highly hydrophilic essence, well‐documented strategies to engineer its crystalline structures, as well as favorable composite capacity with other functional materials.^[^ ^26^, ^27^, ^28^, ^29^, ^30^ ^]^ Especially, by rationally tailoring the ratio of lithium chloride (LiCl) in the PVA hydrogels can enhance their ionic conductivity, low‐temperature resistance, softness, and stretchability, enabling there could be used as electrolyte in the fields of batteries and wearable display equipments.^[^ ^31^, ^32^ ^]^

Here we demonstrate an innovative integrated 3D printing technology for producing personalized FECDs. In addition to the base layer, all materials are printed by direct ink writing (DIW) printer layer by layer without any other processing strategy. In order to realize the printing of multi‐layer electrochromic devices, before printing, we systematically evaluated the 3D printing suitability of N,N′‐bis(3‐sulfonatopropyl)−4,4′‐bipyridinium(SV), N,N′‐bis(3‐sulfonatopropyl)−4,4′‐(thien‐2,5‐diyl)bispyridinium(STV) and N,N′‐bis(3‐sulfonatopropyl)−4,4′‐(3,4‐ethylenedioxylthien‐2,5‐diyl)bispyridinium (SETV) electrochromic inks, electrolyte layer PVA/LiCl ink and encapsulation layer PDMS ink. Different array patterns are designed for the electrochromic functional layer and printed under optimal printing parameters. Subsequently, the electrolyte layer and encapsulation layer are printed sequentially to achieve the integration of electrochromic devices. The FECDs based on hydrogels containing SV, STV, and ETV exhibits outstanding electrochromic properties and strong mechanical properties, including high optical contrast (maximum 54.4% at 360 nm), excellent cycling stability (<5% reduction after 10 000 s) and optical contrast degradation less than 19% after 5000 bending cycles. We further demonstrate the stable applications of this electrochromic device with pattern arrays in wearable electronics, camouflage, and smart windows.

## 2 Result and Discussion

### 2.1 Synthesis and Characterization of Viologen Derivatives

Viologen derivatives SV, STV, and ETV, each containing different thiophene groups and sulfonate substituted with EDOT dibromide and pyridinium borate, are synthesized via Suzuki coupling reaction between thiophene and EDOT dibromide and pyridinium borate (Figure S1, Supporting Information). All viologen derivatives are characterized using ^1^H and ^13^C NMR spectroscopy, confirming their consistency with the proposed structures (Figures S2–S7, Supporting Information). To explore the redox behavior of the viologen derivatives and their potential integration with hydrogels for electrochromic applications, cyclic voltammograms of SV, STV, and ETV (5 mmol L^−1^) are recorded in aqueous LiCl solution (0.1 mol L^−1^) at a scan rate of 100 mV s^−1^ over 1 and 1000 cycles. In the cyclic voltammograms (Figure S9, Supporting Information), two reversible redox pairs are observed, corresponding to the stepwise one‐electron reduction processes of the pyridine moiety in the viologen molecules. Initially, the viologen derivative (V^2+^) is reduced to a radical cation form (V^●+^) with one unpaired electron during the first redox process. Subsequently, the radical cation (V^●+^) undergoes further reduction to an uncharged neutral state (V^0^), forming the second redox pair. Notably, the current density of all viologen derivatives increases linearly with the scan rate (Figure S9a–c, Supporting Information), indicating that the redox reactions are primarily governed by surface‐limited electron transfer kinetics. Additionally, all three viologen derivatives exhibit high electrochemical stability during the 1000‐cycle CV process (Figure S9d–f, Supporting Information), with a slight change in the charge storage capability (Figure S9d–f, Supporting Information).

### 2.2 Design of Integrated 3D Printing Hydrogels‐Based FECDs

**Figure 1:**
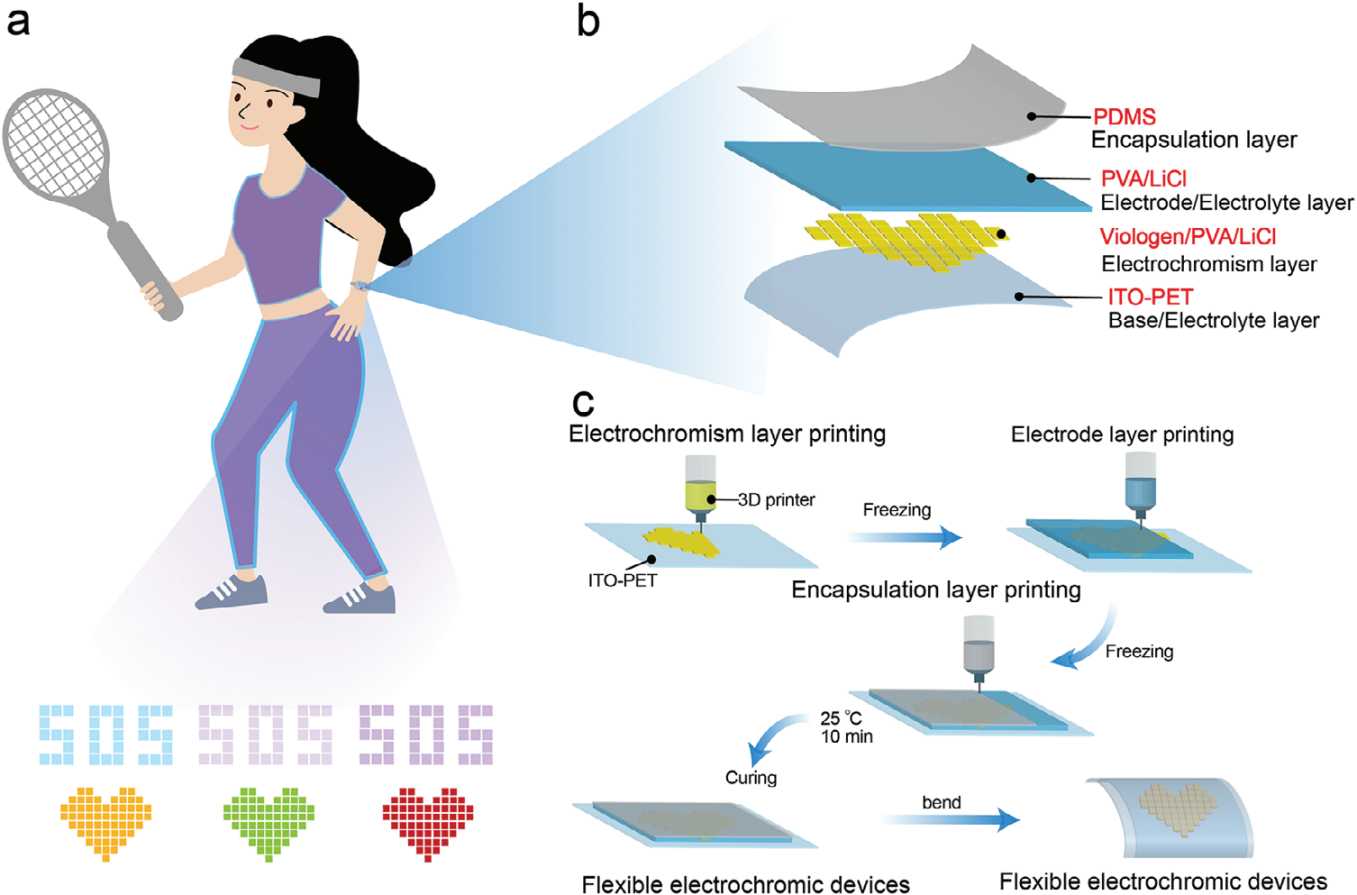
Integrated 3D printing of hydrogels‐based FECDs a) The heart‐shaped array and SOS‐type array are made of ETV hydrogel and STV hydrogel, respectively. b) Structural design of integrated 3D printing of hydrogel‐based FECDs. FECDs consists of a four‐layer stacked structure, which includes encapsulation layer (PDMS), electrolyte layer/electrode layer (PVA/LiCl hydrogel), electrolyte layer (viologen/PVA/LiCl hydrogel) and base/electrode layer (ITO‐PET). c) Integrated 3D printing processes of FECDs. The array of electrochromic layers is printed in three consecutive steps via a multimaterial printing nozzle.

3D printing process is demonstrated to fabricate customized flexible electrochromic devices (FECDs) for wearable electronic applications (**Figure** **1**). The FECDs consist of a four‐layer stacked structure: PDMS as encapsulation layer, PVA/LiCl hydrogel as electrolyte/electrode layer, viologen/PVA/LiCl hydrogel as electrolyte layer, and ITO‐PET as substrate/electrode layer (Figure 1b). Apart from the ITO‐PET layer, other functional layer is printed layer by layer using a DIW printer without any additional processing steps (Figure 1c). Subsequently, arrays of heart‐shaped, SOS‐type, and butterfly patterns are printed sequentially, allowing for the continuous manufacture of customized FECDs using the proposed printing process. Furthermore, the fabricated FECDs can be attachable to the skin of the hand, which can be used in combination with sensing elements or bioelectrode materials to intuitively display biological signals.

### 2.3 3D Printability Evaluation of Multimaterial Electrochromic Inks

**Figure 2:**
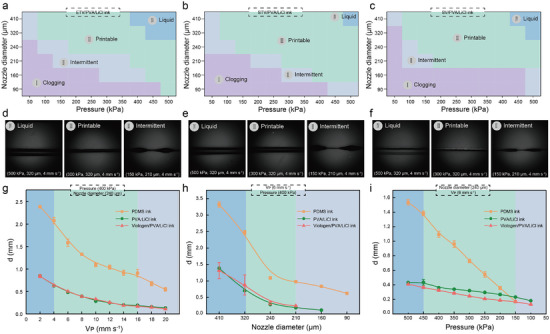
3D printability evaluation of multimaterial electrochromic inks. The phase diagrams show the printability of multimaterial 3D printing inks with electrochromic monomers and PVA/LiCl complexes under different printing parameters (Nozzle diameter: 90–410 µm, Pressure: 50–500 kPa). a) ETV/PVA/LiCl inks, b) STV/PVA/LiCl inks, c) SV/PVA/LiCl inks. The printing area is differentiated into four colors: purple (clogging), gray (intermittent), green (printable) and blue (liquid) based on the extrusion of the ink and the width of the ink. d–f) Physical photographs of the multimaterial inks demonstrating liquid, printable, and intermittent forms under different printing parameters. g) Ink diameter of multilayer electrochromic devices versus printing speed (0–20 mm s^−1^) for the same printing pressure (400 kPa) and nozzle diameter (240 µm). h) Ink diameter of multilayer electrochromic devices versus nozzle diameter (90–410 µm) for the same printing speed (6 mm s^−1^) and printing pressure (400 kPa). i) Ink diameter of multilayer electrochromic devices versus printing pressure (50–500 kPa) for the same nozzle diameter (240 µm) and printing speed (6 mm s^−1^).

The integrated fabrication of multilayer electrochromic devices is a relatively complex and synergistic optimization process. Except for the conductive substrate ITO‐PET layer, the other three layers of the structure (including electrochromic functional layer, electrolyte layer and encapsulation layer) are fabricated using 3D printing technology. Optimization of each electrochromic ink, as well as multilayer structure ink, is necessary to ensure high precision and accurate printing of each device structure layer. To achieve this multi‐material printing, we first characterized the printing parameters of five materials with distinct viscoelastic properties: PDMS ink, PVA/LiCl ink, SV/PVA/LiCl ink, STV/PVA/LiCl ink, and ETV/PVA/LiCl ink. Figure S10 (Supporting Information) demonstrates that all inks exhibit significant shear‐thinning properties, which is essential for smooth extrusion in direct ink 3D printing. Notably, at the same PVA concentration, the apparent viscosity of the composite ink is higher than that of the pure polymer ink. Consequently, incorporating small molecules such as SV, STV, and ETV into PVA markedly increases the modulus and yield stress of the composite ink, thereby ensuring that the printed ink maintains a stable shape during the printing process. Based on the printability evaluation, optimal printing parameters and patterns are designed for the different functional layers to minimize diffusion effects, resulting in high shape fidelity and seamless interface integration. Considering that all three parameters, print air pressure, needle size, and print speed, affect the printing results. To facilitate the optimization of electrochromic layer inks, the print speed is fixed at 10 mm s^−1^, and the print nozzle diameter (90–410 µm) as well as print air pressure (50–500 kPa) are varied to optimize the ETV/PVA/LiCl ink, STV/PVA/LiCl ink and SV/PVA/LiCl ink. It should be noted that all three inks demonstrates different degrees of needle clogging and ink discontinuity at smaller print air pressures and finer print tip ranges. As shown in **Figure** **2**d–f, the ink clogging and discontinuity mainly came from the low air pressures and the inability to make continuous and uninterrupted ink extrusion, which suggests that higher air pressures and larger print tips are needed to ensure the ink extrusion. Therefore, these three inks continued to print by using a large nozzle diameter (>320 µm) and high pressure (>450 kPa), and as expected, at higher pressure ranges and larger needle sizes, the inks printed lines that exceeded the size of the needle, and even exhibited diffusion on the substrate without good shape fidelity, which indicated that optimal printing ranges should be further narrowed. Finally, the three inks are successfully printed in several more moderate needle sizes and pressure ranges.

In order to further explore the printing parameters of multilayer electrochromic device structures. Printed linewidths are quantified and printing parameters are optimized for three inks (viologen/PVA/LiCl ink, PVA/LiCl ink, PDMS ink), when printing speed, nozzle size, and printing pressure are used as single variables. First, the line widths of the three inks at different printing speeds are observed using a microscope at a printing air pressure of 400 kpa and a 240 µm printing nozzle diameter, and the line widths of the three inks are positively correlated with the printing speed. Additionally, the width of printed lines is quantitatively at the same print speed (6 mm s^−1^) and print pressure (400 kPa), the same nozzle diameter (240 µm) and print speed (6 mm s^−1^). As in the experimental group where print speed is the variable, the width of the ink is similarly positively correlated with the needle size and print air pressure. It is noteworthy that the inks for the electrochromic functional and electrolyte layers exhibit relatively consistent printing parameters, which ensures interlayer compatibility during integrated fabrication of multilayer electrochromic devices and does not lead to problems such as shape distortion due to differences in ink parameters.

### 2.4 Integrated 3D Printing of Hydrogels‐Based FECDs

**Figure 3:**
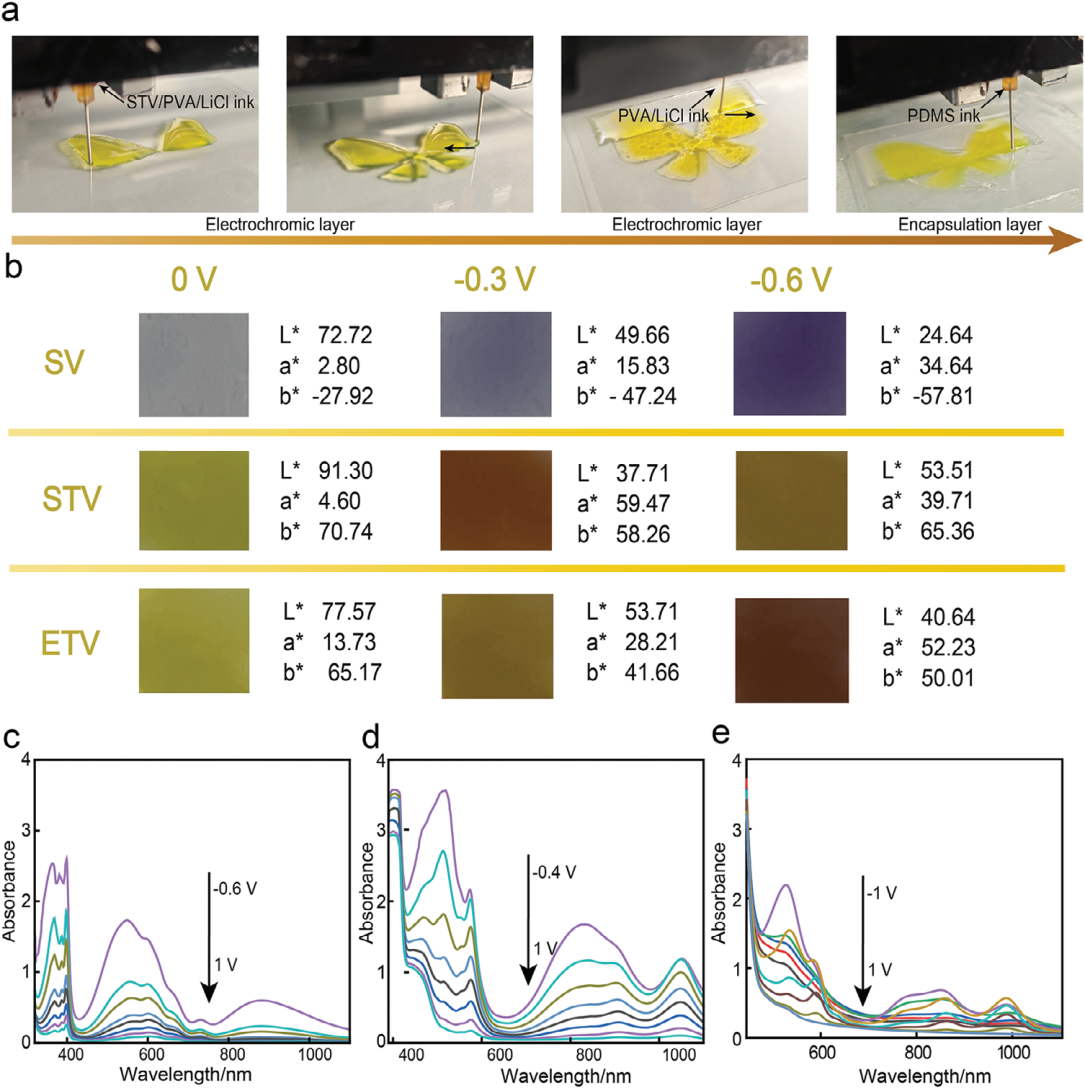
3D Printing of integrated hydrogels‐based FECDs and electrochromic properties. a) Sequential snapshots of 3D‐printed multilayer electrochromic device with three different inks. PDMS ink for the encapsulation layer (dimensions: 40 mm × 40 mm), STV/PVA/LiCl ink for the electrochromic layer, and PVA/LiCl ink for the electrolyte layer (dimensions: 35 mm × 30 mm). b) Photographs (dimensions: 5 mm × 5 mm) and parameters of the SV, STV and ETV hydrogel‐based FECDs color upon applying different potentials. c) Spectroelectrochemistry for FECDs based on SV hydrogel. The applied potential was increased in 0.1 V increments from −0.6 V to 1 V. d) Spectroelectrochemistry for FECDs based on STV hydrogel. The applied potential was increased in 0.1 V increments from −0.4 V to 1 V. e) Spectroelectrochemistry for FECDs based on ETV hydrogel. The applied potential was increased in 0.1 V increments from −1 to 1 V.

Based on the systematic evaluation of the printability of multimaterial electrochromic inks and electrolyte inks, and encapsulation layer inks, we select the optimal 3D printing parameters (pressure: 300 kPa, speed: 6 mm^−1^, and needle: 240 µm) for the subsequent in printing of the multi‐layer electrochromic devices in order to achieve high printing fidelity. Diversified print patterns, including SOS arrays, heart arrays, and different animal shapes, are designed according to the application scenarios of health monitoring and anti‐counterfeiting. For the electrochromic functional layer, we take the ETV/PVA/LiCl ink as an example and print a heart‐shaped array on the ITO‐PET substrate, as shown in **Figure** **3**a. The optimized parameters can print the pattern with high fidelity even for small‐size, high‐density electrode arrays. When the printing of the electrochromic functional layer is completed, the cross‐linking of the gel needs to be completed after 2–3 cyclic freeze‐thaw experiments under two temperature regimes of −20 and 25 °C. Subsequently, the electrolyte layer is printed on the electrochromic functional layer. The electrolyte layer serves to ensure that the device maintains a complete conductive pathway during energization and ensures stable discoloration of the device, so we print a rectangle with an area larger than that of the functional layer on this layer, so that all of the array units are connected in series on a conductive pathway. Similarly, this layer of the structure is subjected to 2–3 cycles of freeze‐thawing to cross‐link the gel network. Finally, in order to prevent water evaporation from the gel structure of each layer of the electrochromic device during use, we print a PDMS elastomer layer on the topmost layer. Like the electrolyte layer, we print a rectangular structure for the PDMS encapsulation layer. It should be noted that the encapsulation layer is much larger than the functional layer and the electrolyte layer because a larger encapsulation area is more effective in preventing the evaporation of water and keeping the device's performance stable and long‐lasting.

We first measure the spectroelectrochemistry of the three FECDs by connecting copper foil to both electrodes and K₄Fe(CN)₆ to the electrochromic layer as additional redox components (Figure 3c–e; Figure S11, Supporting Information). In the absence of external voltage, the three devices show low absorption values across the entire visible light region. The colors of the FECDs based on SV, STV, and ETV hydrogels are colorless, yellow‐green, and yellow, respectively (Figure 3b). At this stage, the three viologen molecules are maintained in the V^2^⁺ state. When a potential is applied to the viologen hydrogel‐based FECDs, each device undergoes a distinct color transition. This electrochromic behavior stems from the production of radical cations following single‐electron reduction, with the color alteration being a result of the elevated concentration of these singly reduced radical cations.^[^ ^33^ ^]^ When a −0.3 V voltage is applied, the visible absorption spectrum changes significantly. Two characteristic absorption peaks are detected at 360, 580, 510, 800, 500, and 850 nm for FECDs based on SV, STV, and ETV hydrogels, respectively. Visually, the three FECDs appear light purple, reddish brown, and light brown. This color change is due to the purple pigment diionic radical cation undergoing π*–π* and n–π* transitions to generate single ion radicals. As the applied voltage increases, the concentration of single ion radicals increases, and the absorbance increases significantly. When a −0.6 V voltage is applied, the three FECDs appear dark purple, light brown, and dark brown, respectively, due to the free conversion of the violet single cation to the neutral state.

### 2.5 Electrochromic and Mechanical Properties of Integrated 3D‐Printed Hydrogels‐Based FECDs

**Figure 4:**
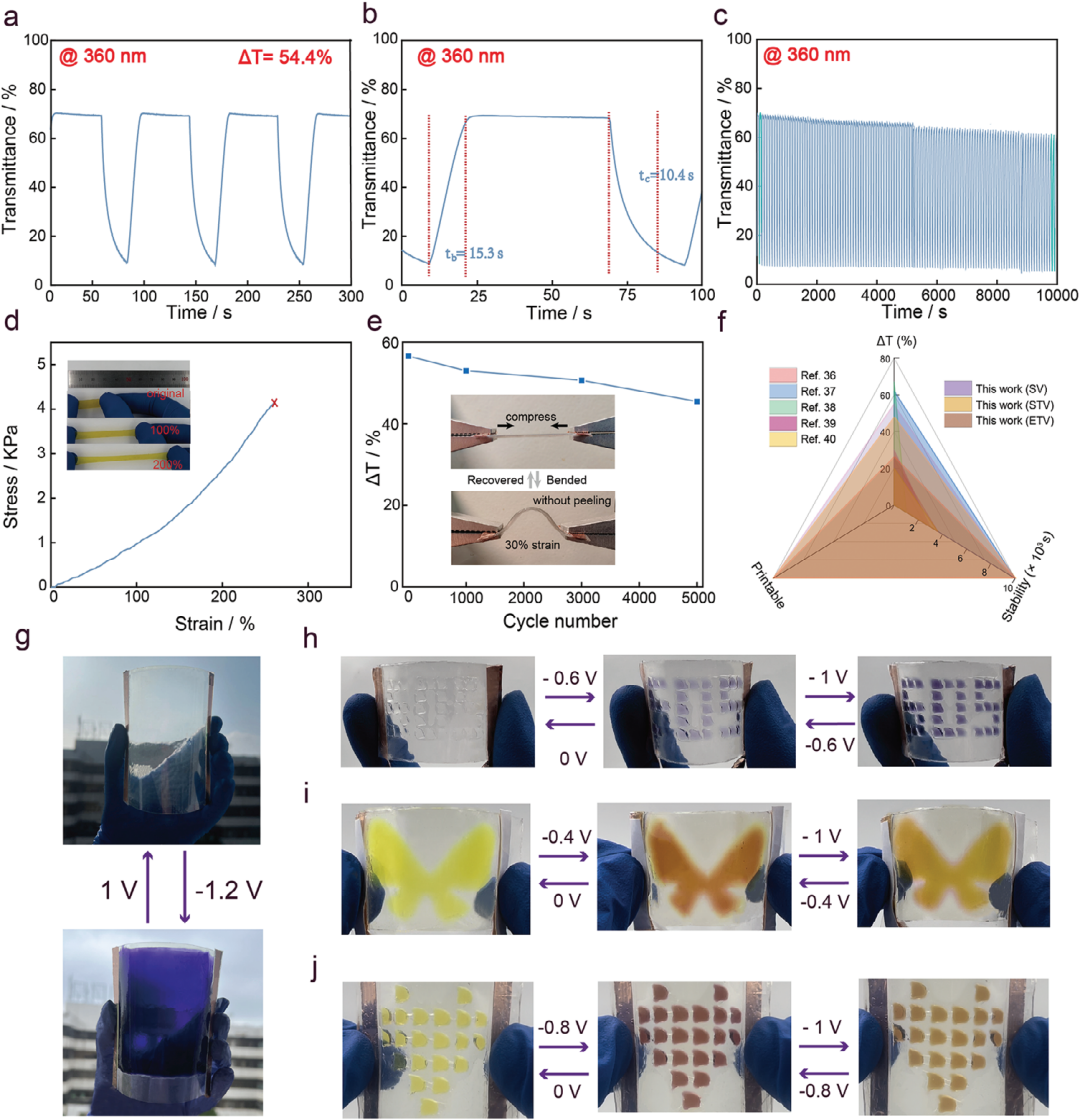
Electrochromic and mechanical properties of integrated 3D‐printed hydrogels‐based FECDs. a) Time‐transmittance profiles of the FECDs (dimensions: 10 mm × 10 mm)containing SV hydrogel at 360 nm. b) Measurement of response times from the transmittance spectra of the FECDs (dimensions: 10 mm × 10 mm) containing SV hydrogel. c) Transmittance profiles of the FECDs (dimensions: 10 mm × 10 mm) containing SV hydrogel against continuous long term switching at 360 nm. d) Pressure‐strain profiles of an ETV hydrogel (dimensions: 40 mm × 8 mm × 1 mm). The inset shows a photograph of the ETV hydrogel hydrogel before and after stretching. e) The transmittance at 360 nm of the FECDs (dimensions: 25 mm × 15 mm) containing SV hydrogels were measured after 5000 cycles of long‐term mechanical bending with a strain of 30%. f) Comparison of integrated 3D‐printed hydrogels‐based FECDs with previously reported typical viologen FECDs. g) Photographs of a 10 × 10 cm^2^ integrated 3D‐printed FECDs (dimensions: 110 mm × 100 mm) containing SV hydrogel. h) Photographs of a SOS array (dimensions: 3 mm × 3 mm) FECDs containing SV hydrogel (dimensions: 45 mm × 25 mm) under application of different voltages. i) Photographs of a butterfly pattern FECDs containing SV hydrogel (dimensions: 35 mm × 30 mm) under application of different voltages. j) Photographs of a heart‐type array (dimensions: 3 mm × 3 mm) FECDs containing SV hydrogel (dimensions: 30 mm × 30 mm) under application of different voltages.

To further evaluate the electrochromic properties of 3D‐printed hydrogel‐based FECDs, we conduct comprehensive kinetic studies, including measurements of transmittance changes (ΔT), response time, and cycling stability at different wavelengths. The maximum transmittance changes for the SV, STV, and ETV hydrogel‐based FECDs are 54.4% (360 nm) and 33.21% (580 nm), 27.13% (510 nm) and 47.6% (800 nm), 25.75% (500 nm) and 17.79% (850 nm) (**Figure** **4**a; Figure S12, Supporting Information). The response times for FECDs based on hydrogels containing SV, ATV, and ETV to reach over 90% of their maximum optical contrast during a switching time are measured to be 25.7 s (360 nm) and 31.9 s (580 nm), 28.1 s (510 nm) and 52.2 s (800 nm), 22 s (500 nm) and 31.2 s (850 nm). respectively. Interestingly, all three FECDs demonstrate excellent cycling stability over a test period exceeding 10 000 s. Notably, the FECDs utilizing π‐extended viologens exhibit exceptional stability (Figure S12, Supporting Information). This remarkable stability can be attributed to the inhibition of radical species dimerization by isolating the viologen moiety, a phenomenon well‐documented in existing literature.^[^ ^34^, ^35^, ^36^ ^]^

To fabricate FECDs, it is essential to ensure that each component of the device exhibits good flexibility. The viologen derivatives are synthesized in this study, which have enhanced solubility in water, are incorporated into a PVA hydrogel matrix to create the electrochromic layer. The 3D‐printed hydrogel‐based ETV, measuring 3 cm in length, can be stretched reversibly to 9 cm, demonstrating excellent flexibility (Figure 4d). SV/PVA/LiCl and STV/PVA/LiCl prepared using the same method also exhibit outstanding flexibility (Figure S13a, Supporting Information). Other parts of the FECDs, including PDMS and PVA/LiCl hydrogel, also display exceptional stretchability (Figure S13a,b, Supporting Information). In addition, stretchable transparent electrolytes and electrodes are indispensable components. In this work, PVA hydrogel containing LiCl was chosen as the ideal electrolyte and electrode material (Figure S13c, Supporting Information). This hydrogel can stretch up to three times its original length, indicating that PVA/LiCl hydrogel possesses excellent elasticity. The transmittance PVA hydrogel maintains a consistent transmittance of 90% across the wavelength range of 300–1100 nm (Figure S13d, Supporting Information), suggesting that the synthesized hydrogels have sufficient tensile strength and transparency to function effectively as both electrodes and electrolytes. The optical contrast of the device was evaluated under different bending cycles (Figure 4), showing that after 5000 bending cycles, the optical contrast only decreased by 19%. The cross‐section during bending (Figure 4e) reveals that all parts of the device remain tightly connected when significantly bent. As shown in Figure 4f, compared to typical viologen FECDs,^[^ ^37^, ^38^, ^39^, ^40^, ^41^, ^42^ ^]^ 3D‐printed hydrogel‐base viologen FECDs exhibits superior overall performances especially including excellent optical contrast as well as outstanding stability against pulse switching cycling. The advantageous electrochromic performance and 3D printability of these devices provide promising technical support and a theoretical basis for the large‐scale and patterned production of FECDs.

Finally, we integrate 3D‐printed FECDs with various patterns and sizes to suit different application scenarios. As illustrated in Figure 4g, the hydrogel FECDs fabricated through our 3D printing design offer ease of preparation and scalability. To assess their scalability, we fabricate smart windows measuring 10 × 10 cm^2^ based on these FECDs. The device exhibits a uniform color change from colorless to dark purple when a voltage of −1.2 V is applied. Additionally, we create FECDs with patterned letters composed of small squares (3 mm × 3 mm) (Figure 4h). While the patterns could not be perceived in the bleached state, the letters appeared in deep purple at −1 V. When we applied 1 V, the FECDs return to the bleached state and the patterns disappeared. In addition, a STV hydrogel‐based FECDs device with a butterfly shape was prepared (Figure 4i), and the color changed from yellow to brown and then to orange when the voltage changed from −1 to 1 V. Similarly, FECDs with a cardioid pattern, also composed of small squares (3 mm × 3 mm) (Figure 4j), show color changes from yellow to orange to red when the voltage varies from −1 to 1 V. Moreover, this reversible electrochromic behavior is maintained even after bending. These findings highlight the versatility and robustness of our 3D‐printed hydrogel‐based FECDs, demonstrating their potential for diverse applications in wearable electronics and smart windows.

### 2.6 Integrated 3D‐Printed Hydrogels‐Based FECDs for Wearable Displays and Camouflage Applications

**Figure 5:**
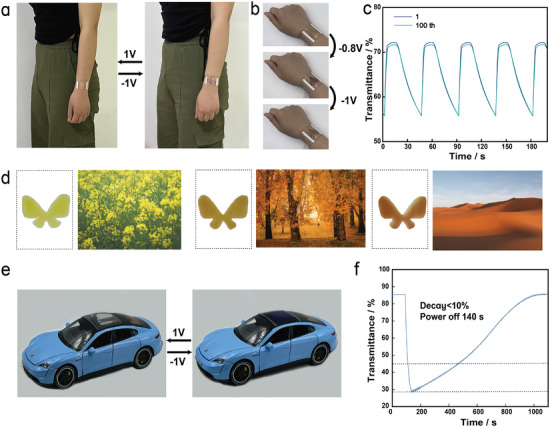
Integrated 3D‐printed hydrogels‐based FECDs for wearable displays and camouflage applications. a) Schematic diagram of the FECDs worn on the wrist. The electrochromic layer of the FECD (dimensions: 45 mm × 25 mm) is an SOS array containing SV hydrogel. b) Demonstration of a color‐changing on‐skin tattoo based on FECDs (dimensions: 30 mm × 30 mm) containing ETV hydrogel. c) Transmittance of continuous long‐term switching at 850 nm of cardioid array FECDs containing ETV hydrogel. d) Application scenarios of the FECDs containing STV hydrogel in adaptive camouflage, the rapeseed fields, maple forests, and desert photos are provided under the Pexels free use agreement and may be used free of charge in accordance with the terms of the agreement. e) Application scenarios of the FECDs (dimensions: 30 mm × 30 mm) based on SV hydrogels in smart windows. f) Transmittance of FECDs based on SV hydrogels at 360 nm under −1 V for 30 and power off for 970 s.

Based on the excellent electrochromic performance and mechanical properties of the integrated 3D‐printed FECDs, we further demonstrate their practical application in wearable electronic devices through cardioid and SOS arrays (**Figure** **5**a). The square grid (1 mm × 1 mm) in the SOS array can reversibly change from colorless to purple at a low driving potential of −1.0/1.0 V, mimicking emergency signals. Using the 3D‐printed heart‐shaped array, we observe that the square mesh (3 mm × 3 mm) can reversibly change color from yellow to orange to red at a low driving potential of −1.0/1.0 V, thereby imitating changes in heart rate. Additionally, the FECDs can operate under various mechanical deformations, such as bending, without affecting their electrochromic properties (Figure 5b). Furthermore, the transmittance of the heart‐shaped array FECDs containing ETV hydrogel decreases by only 19% after 100 color cycles at 850 nm, demonstrating good durability (Figure 5c). These results underscore the potential of 3D‐printed hydrogel‐based FECDs for advanced wearable electronic technologies.

The application of integrated 3D‐printed FECDs in camouflage displays is shown in Figure 5d. When different voltages are applied, different colors are presented. Adaptive camouflage systems operating in the visible light spectrum have broad utility across various domains. FECDs incorporating STV can display three distinct reversible colors with minimal voltage, closely resembling natural hues of flowers (yellow), autumn (orange), and desert (red). Using STV hydrogel‐based FECDs in clothing allows for effective concealment in diverse natural environments, demonstrating impressive camouflage capabilities.

Finally, we demonstrate the application of SV hydrogel‐based FECDs in smart car windows (Figure 5e). The device remains highly transparent without any applied voltage. Tinted states offer various colors and a degree of privacy. When the outdoor temperature is high, applying −1 V for several seconds turns the smart window dark purple, blocking solar radiation and keeping the interior cool. Furthermore, with only 30 s of potential pulse stimulation at −1 and 1 V, the FECDs can maintain its color for about 140 s without any electrical input (with transmittance decreasing by less than 10%) (Figure 5f). This excellent optical memory effect can reduce the energy consumption of hydrogel‐based FECDs, making them advantageous for various practical applications in smart windows.

## 3 Conclusion

In this study, we demonstrate a 3D printing process through continuous multi‐material printing of FECDs. The 3D printability of the electrochromic layer, electrolyte layer, and encapsulation layer ink are evaluated while reasonable printing parameters are selected. At the same time, the electrochromic array pattern designs realize the manufacturing of multi‐layer flexible electrochromic devices. The results demonstrate that the 3D printing‐based FECD exhibits exceptional electrochromic properties and robust mechanical characteristics, including high optical contrast, excellent cycling stability, and a decrease in optical contrast of less than 19% after 5000 bending cycles. Furthermore, this study showcases the stable application of this electrochromic device with pattern array in wearable electronics, camouflage, and smart windows. Overall, the proposed integrated 3D printing technology provides a promising platform for simplified and customized manufacturing of high‐performance flexible electrochromic devices for various applications such as wearable devices, camouflage, and smart windows.

## 4 Experimental Section

### Materials

All chemicals and reagents were procured from commercial suppliers and utilized without further purification. 2,5‐Dibromothiophene, 4,4′‐bipyridine, 4‐pyridineboronic acid pinacol ester, 2,5‐dibromo‐3,4‐ethylenedioxythiophene, HCl, NaOH, tetrakis(triphenylphosphine)palladium(0), potassium bromide, 1,4‐dioxane, DMF, anhydrous acetonitrile, potassium phosphate, 1,3‐propanesultone, poly(vinyl alcohol) (Mw 146–186 kDa), potassium ferrocyanide, PDMS were purchased from Dow Corning, and lithium chloride were purchased from Aladdin. ITO‐PET (<10 Ω sq^−1^) were purchased from *Zhuhai Kaivo*.

### Preparation of Multimaterial Precursor Ink

*Electrochromic functional layer ink*: LiCl (1 g), the viologen derivative (5 mmol L^−1^), K_4_Fe(CN)_6_ (0.018 g), PVA powder (1 g) was dissolved in 8 mL deionized water. Then, the mixture was vigorously stirred at 90 °C for 2 h to yield a homogeneous solution. The resulting solution was then deaerated using a centrifugal mixer at 8000 rpm for 10 min to produce the ink.

*Electrolyte layer ink*: LiCl (1 g), PVA powder (1 g) was dissolved in 8 mL deionized water. Then, the mixture was vigorously stirred at 90 °C for 2 h to yield a homogeneous solution. The resulting solution was then deaerated using a centrifugal mixer at 8000 rpm for 10 min to produce the ink.

*Encapsulation layer ink*: PDMS ink can be obtained by mixing the PDMS precursor solution and curing agent in a ratio of 10:1 and stirring under mechanical agitation for 20 min to make the mixture homogeneous. It should be noted that to prevent PDMS from curing, this ink needs to be ready to use.

### 3D‐Printed Multilayer Electrochromic Devices

The optimization of multimaterial inks and the 3D printing of multilayer electrochromic devices was realized on a direct ink 3D printer (DB 100, Shanghai MiFang Electronic Technology Co., Ltd., Shanghai, China). Several sizes of 90, 160, 210, 240, 320, and 410 needles were used in the printing process, and the printing air pressure was regulated from 0 to 500 kPa and the printing speed from 2–20 mm s^−1^. The modeling of the 3D‐printed layers was drawn using Adobe Illustrator 2022 software and saved in SVG format. Before printing, the 3D model in SVG format was imported into the DB 100 software, where further adjustments could be made to the model, and the printing parameters could be adjusted to start printing. After printing, the electrochromic layer and the electrolyte layer were freeze‐thawed for several cycles at temperatures ranging from −20– 25 °C to cross‐link the gel network.

### Optical Characterization

To assess the optical characteristics of viologen derivatives, UV–vis absorption spectra was examined in H_2_O (Figure S8, Supporting Information).

### Electrochemical Properties

Electrochemical assessments were conducted utilizing a Versa Stat 3 electrochemical workstation (EG&G Princeton Applied Research). The redox characteristics of the viologen derivatives were scrutinized via cyclic voltammetry analyses employing a standard three‐electrode configuration. Glassy carbon, Ag/AgCl, and Pt wire were designated as the working, reference, and counter electrodes, respectively. These experiments were carried out in an aqueous solution containing LiCl (0.1 m) under a nitrogen atmosphere, with varying scanning speeds.

### Mechanical Measurement

A universal testing machine (ZQ‐990LB, ZHIQU Precision Instrument) was employed to conduct uniaxial tensile tests on hydrogel samples (dimensions: 40 mm (L) × 8 mm (W) × 1 mm (T)) at room temperature, utilizing a tensile speed of 500 mm min^−1^. For cyclic tensile testing of electrochromic devices, a test speed of 50 mm min^−1^ was utilized.

### Electrochromic Characterization

Spectroelectrochemistry and electrochromic kinetic studies of the devices were systematically investigated by combining a SPECORD 200 PLUS UV–vis spectrophotometer and a Versa Stat 3 electrochemical workstation (EG&G Princeton Applied Research). The ITO‐PET/PVA hydrogel/PDMS device was used as a blank reference during testing.

### Statistical Analysis

Origin 2018 software was used to assess the statistical significance of all comparative studies and all results were expressed as mean ± standard deviation (SD). The data distribution of all parameter tests was assumed to be normal, but no formal tests were performed, and no significant difference analysis was performed.

## Conflict of Interest

The authors declare no conflict of interest.

## Author Contributions

X.L., R.W., and Z.Z. contributed equally to this work. X.L. reviewed and edited the final manuscript and wrote the original draft, and performed software, formal analysis, data curation, experiment, and conceptualization. R.W. reviewed and edited the final manuscript and wrote the original draft, and performed experiment, formal analysis, and data curation. Z.Z. performed experiment and data curation. M.S. performed experiment. L.Y. performed experiment. J.X. performed funding acquisition. H.Y. performed experiment and conceptualization. B.L. reviewed and edited the final manuscript and wrote the original draft, acquired resources, and performed supervision, data curation, conceptualization, and funding acquisition.

## Supporting information

Supporting Information

## Data Availability

The data that support the findings of this study are available in the supplementary material of this article.
