# Supplementary material for: 3D‐Printed Hydrogel‐Based Flexible Electrochromic Device for Wearable Displays
Source: Adv Sci (Weinh). 2024 Aug 9;11(38):2404679. doi: 10.1002/advs.202404679 (PMC11481265; doi:10.1002/advs.202404679)
Supplement: Supplementary file 1 — Supporting Information [file ADVS-11-2404679-s001.docx]

**Supporting information**

**3D-Printed hydrogel-base Flexible Electrochromic Device for Wearable Displays**

Xiaoyu Luo^1,2#^, Rongtai Wan^1,2#^, Zhaoxian Zhang^1,2#^, Manting Song^1,2^, Lixia Yan^1,2^, Jingkun Xu^1,3^*, Hanjun Yang^1,2^*, Baoyang Lu^1,3^*

^1^ Flexible Electronics Innovation Institute, Jiangxi Key Laboratory of Flexible Electronics, Jiangxi Science and Technology Normal University, Nanchang 330013, Jiangxi, PR China

^2^ School of Pharmacy, Jiangxi Science and Technology Normal University, Nanchang 330013, Jiangxi, PR China

^3^ School of Chemistry and Chemical Engineering, Jiangxi Science and Technology Normal University, Nanchang 330013, Jiangxi, P. R. China

^4^ School of Chemistry and Materials Science, East China University of Technology, Nanchang 330013, Jiangxi, PR China

* Corresponding author. Tel: +86-791-88537967; +86-791-83823320.

Email: xujingkun1971@yeah.net; yanghj@jxstnu.edu.cn; luby@jxstnu.edu.cn

The authors contribute equally to this work.

**Supporting information**

**Contents**

**Figure S1.** Synthetic routes of SV, STV, and ETV

**Figure S2.** ^1^H NMR of SV

**Figure S3.** ^13^C NMR of SV

**Figure S4.** ^1^H NMR of STV

**Figure S5.** ^13^C NMR of STV

**Figure S6.** ^1^H NMR of ETV

**Figure S7.** ^13^C NMR of ETV

**Figure S8.** UV-vis absorption of SV, STV and ETV

**Figure S9.** Electrochemical properties of viologen derivatives. Cyclic voltammograms (CVs) of 0.1 mmol L^-1^ SV (a), STV (b), and ETV (c) in H_2_O at different scan rates; Long-term CVs of the deposited SV (d), STV (e), and ETV (f) film at a constant potential scan rate of 100 mV s^-1^

**Figure S10.** Rheological properties of multimaterial inks. (a) Apparent viscosity as a function of shear rate for multimaterial inks. (b) Shear storage modulus as a function of shear angle frequency.

**Figure S11.** The transmittance spectra of FECDs based on SV (a), STV (b), and ETV (c).

**Figure S12.** Electrochromic properties of integrated 3D printed hydrogels-based FECDs. Time-transmission curve of FECDs based on SV (a, b), STV (d, e), and ETV (g, h) at different wavelengths; Transmittance profiles of FECDs based on SV (c), STV (f), and ETV (i) against continuous long-term switching at different wavelengths.

**Figure S13.**  (a) Pressure-strain profiles of the SV, STV and PVA/LiCl hydrogel. (b) Pressure-strain profiles of the PDMS. (c) Photographs of a PVA hydrogel before and after stretching. (d) Transmittance spectra of a blank PVA hydrogel and a piece of ITO-PET. The inset shows a photograph of a blank PVA hydrogel.

**Materials and Methods**

(1) Synthesis of viologen derivatives

Synthesis of N, N′-Bis(3-sulfonatopropyl)-4,4′-bipyridinium (SV). SV was synthesized according to a reported literature procedure.[**1]** ^1^H NMR (400 MHz, D_2_O) *δ* (ppm): 9.12 (d, J = 6.4 Hz, 1H), 8.53 (d, J = 6.3 Hz, 1H), 4.85 (t, J = 7.4 Hz, 1H），2.99（t, J = 7.3 Hz, 1H），2.49（p, J = 7.4 Hz, 1H）.^13^C NMR（100MHz, D_2_O）*δ*（ppm）：150.36, 145.74, 127.21, 60.24, 47.07, 26.19.

Synthesis of N, N′-Bis(3-sulfonatopropyl)-4,4′-(thien-2,5-diyl)-bispyridinium (STV). STV was synthesized according to a reported literature procedure.**[2]** ^1^H NMR (400 MHz, D_2_O) *δ* (ppm): 8.78 (d, J = 6.5 Hz, 1H), 8.26 (d, J = 6.4 Hz, 1H), 8.05 (d, J = 4.2 Hz, 1H), 4.70-4.63 (m, 2H), 2.94 (t, J = 7.2 Hz, 1H), 2.41 (p, J = 7.4 Hz, 1H). ^13^C NMR (100 MHz, D2O)*δ*(ppm)：148.29, 144.63, 142.80, 132.99, 123.72, 59.23, 47.09, 26.08.

Synthesis of N, N′-Bis(3-sulfonatopropyl)-4,4′-(3,4-ethylenedioxylthien-2,5-diyl)bispyridinium (ETV). ETV was synthesized according to a reported literature procedure.[2] ^1^H NMR (400 MHz, D_2_O) δ 8.27 (d, *J* = 6.5 Hz, 3H), 7.82-7.72 (m, 3H), 4.20 (t, *J* = 7.4 Hz, 3H), 4.11 (s, 3H), 2.59-2.44 (m, 3H), 1.94 (d, *J* = 7.5 Hz, 1H).^13^C NMR (101 MHz, D_2_O) δ 146.81, 145.75, 143.96, 140.87, 123.12, 115.84, 65.38, 59.01, 47.94, 47.17, 26.04.


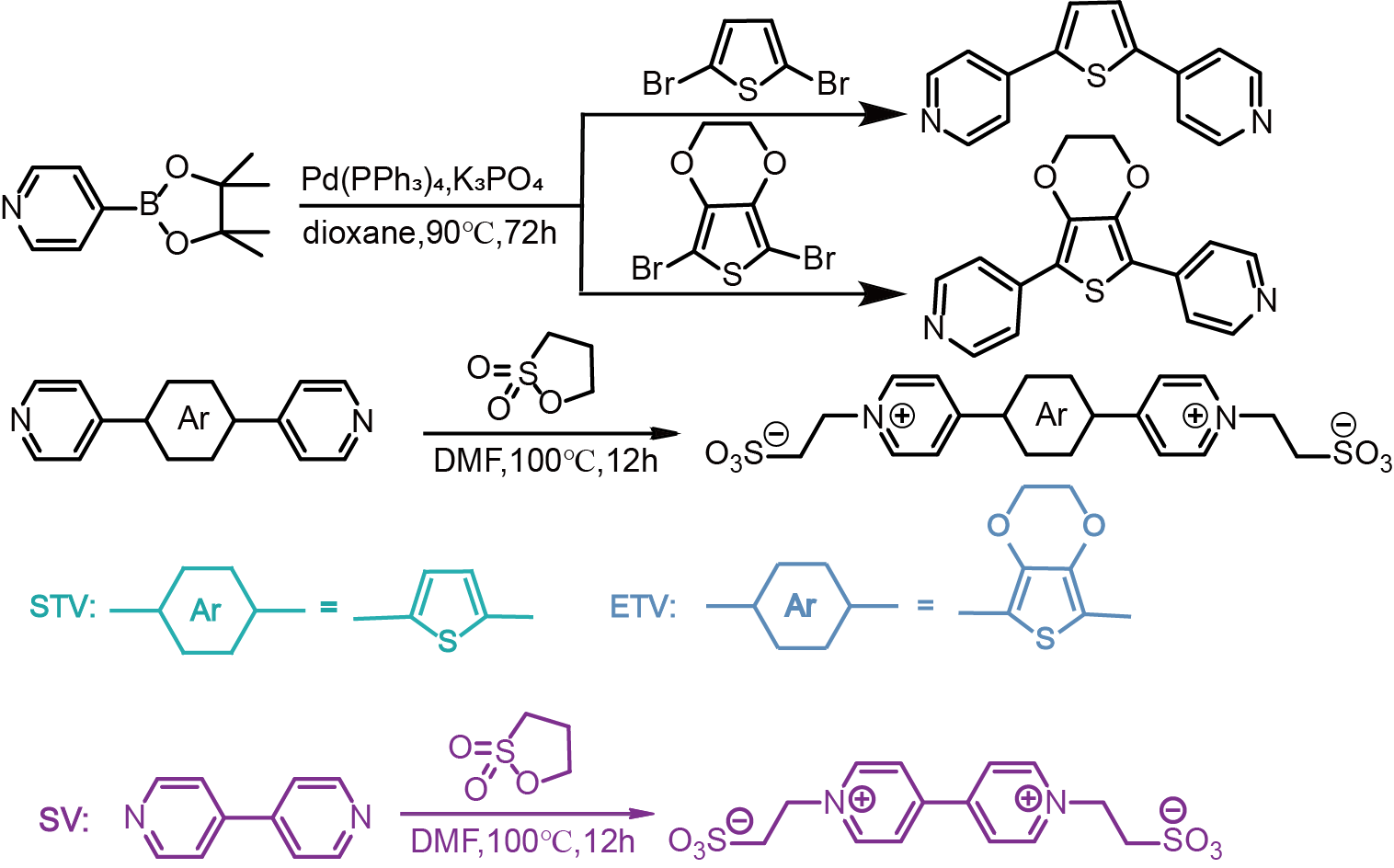


**Figure S1.** Synthetic routes of SV, STV, and ETV


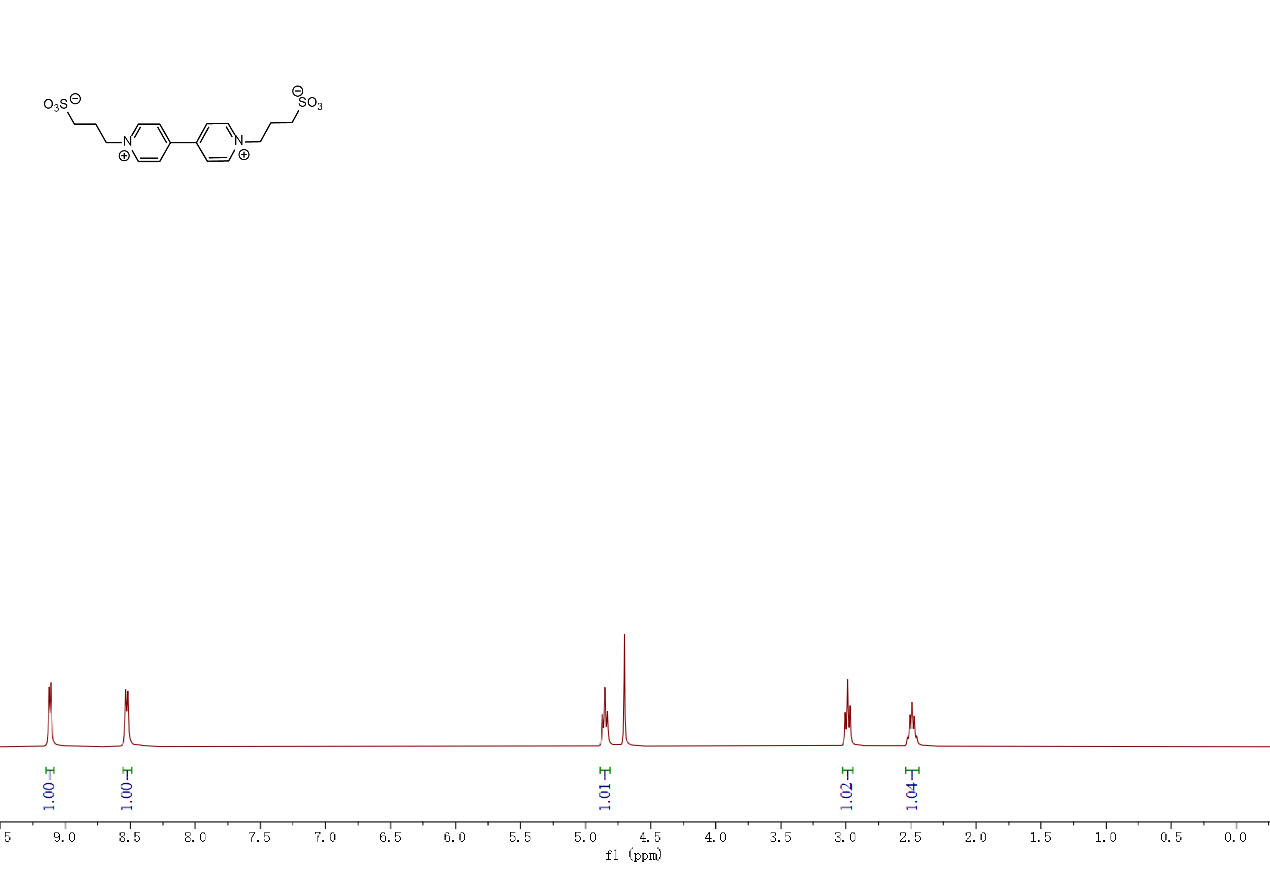


**Figure S2.** ^1^H NMR of SV.


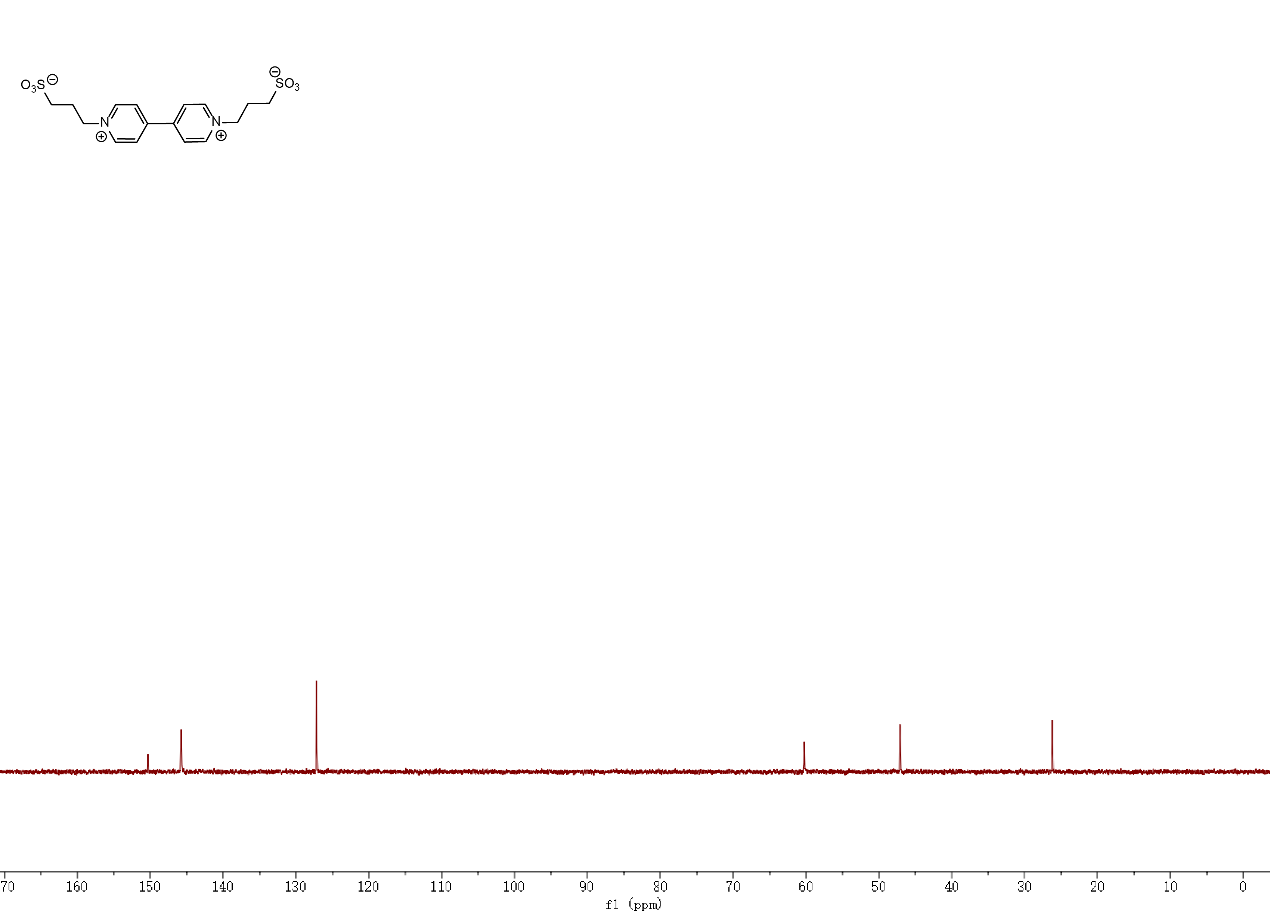


**Figure S3.** ^13^C NMR of SV.


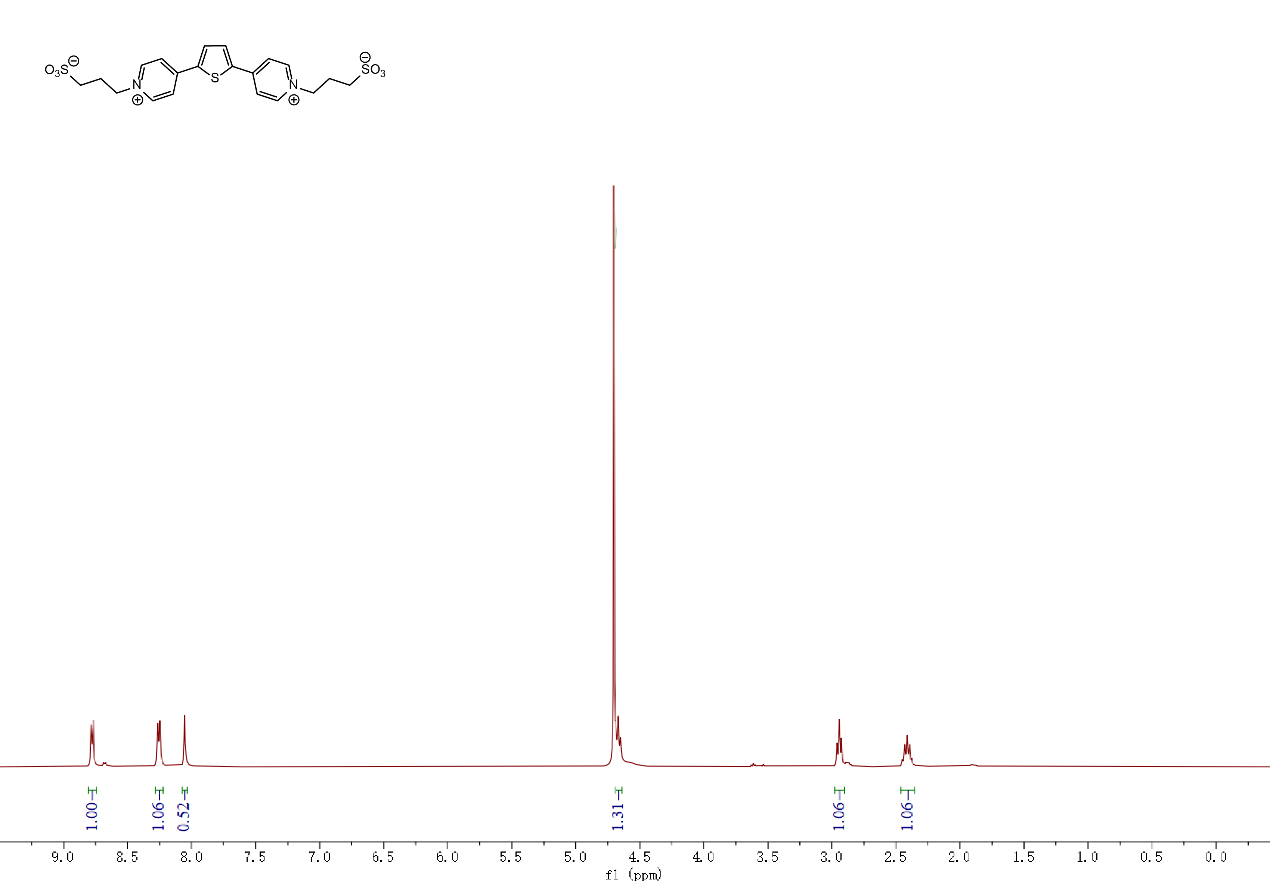


**Figure S4.** ^1^H NMR of STV.


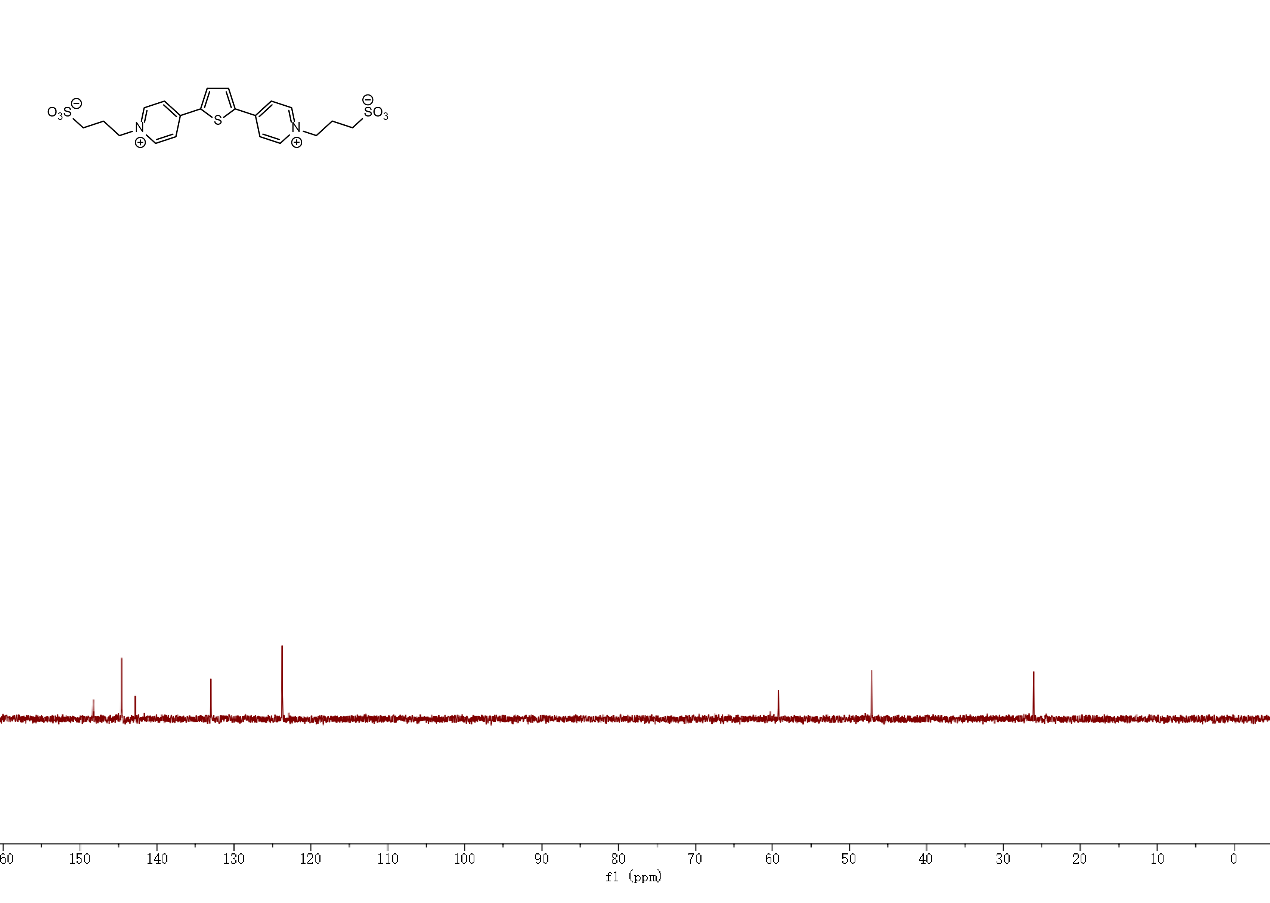


**Figure S5.** ^13^C NMR of STV.

**
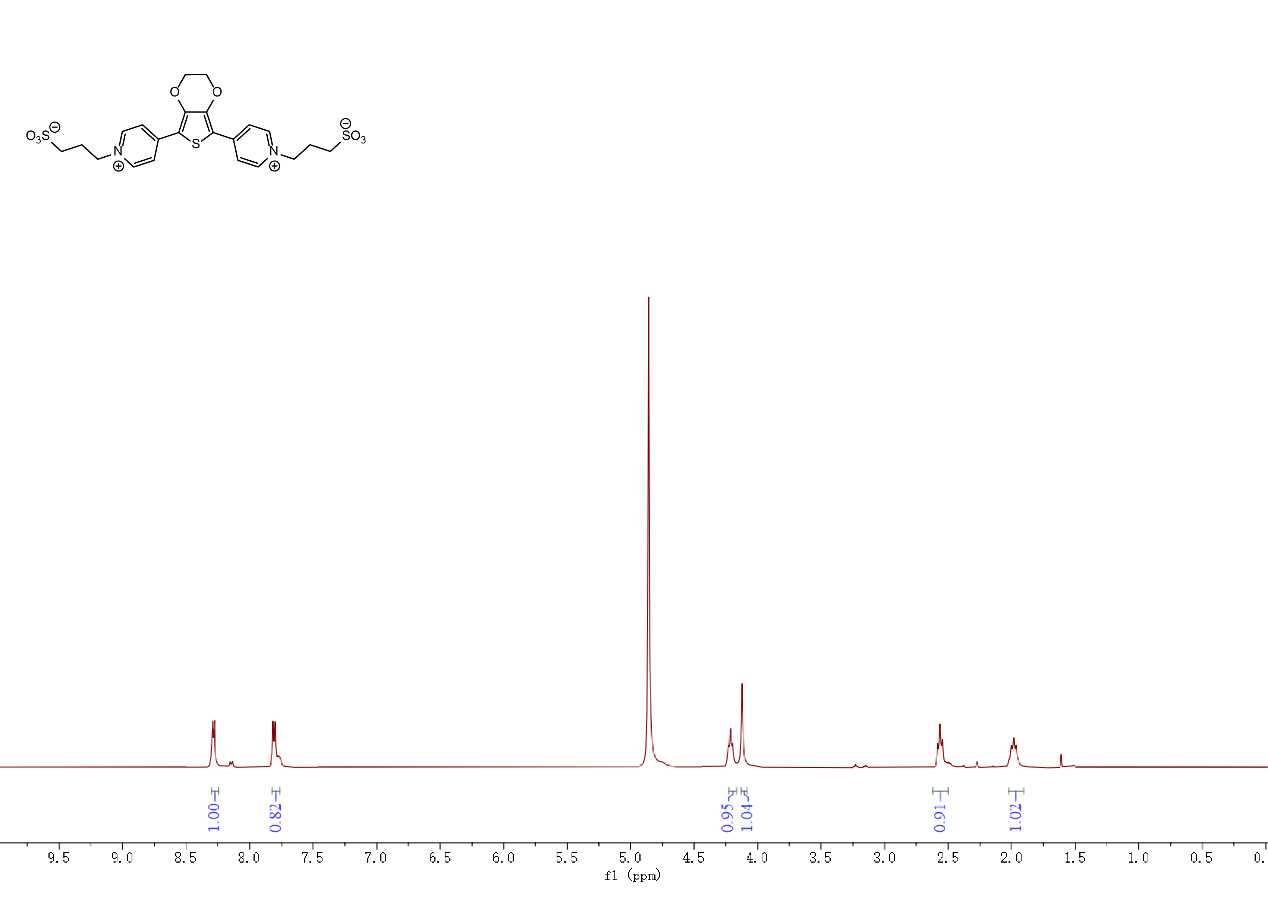
**

**Figure S6.** ^1^H NMR of ETV.


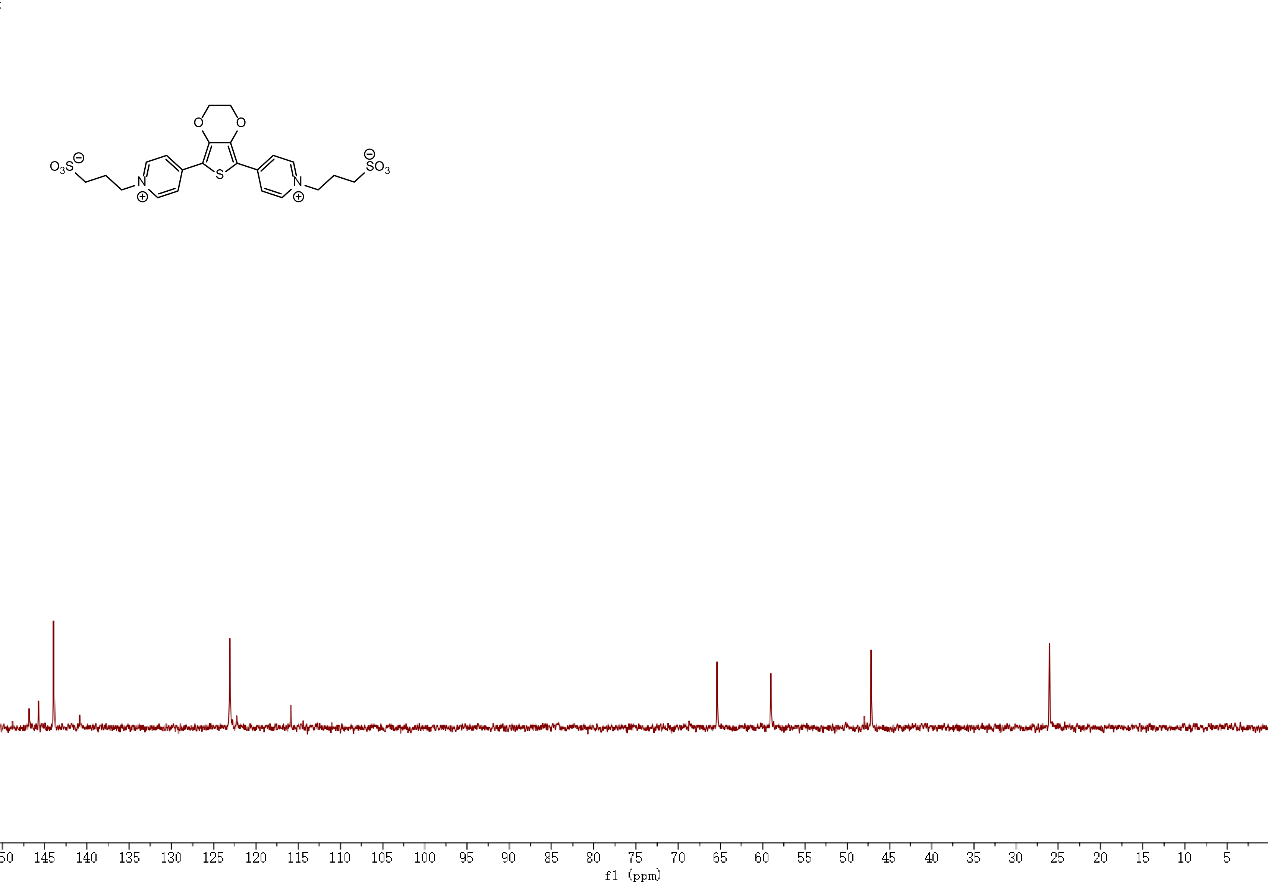


**Figure S7.** ^13^C NMR of ETV.

(2) Optical characterization

To assess the optical characteristics of viologen derivatives, we examine their UV-vis absorption spectra in H_2_O (Figure S8). The maximum absorption peak of STV and ETV occurs at 375 nm and 414 nm, they appear red-shifted by more than 110 nm compared to SV. This significant red-shift phenomenon is mainly attributed to the strong intramolecular charge transfer interaction between the electron-donating thiophene group and the electron-withdrawing pyridinium cation.


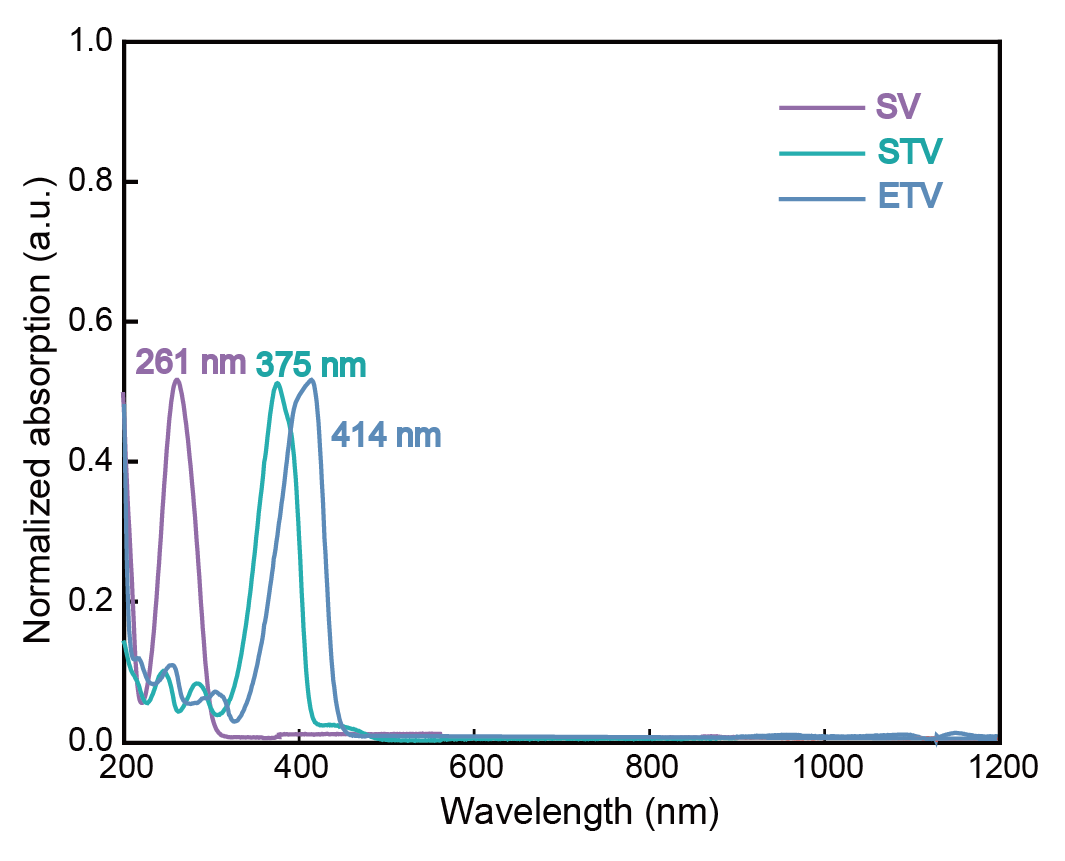


**Figure S8.** UV−vis absorption of SV, STV and ETV.


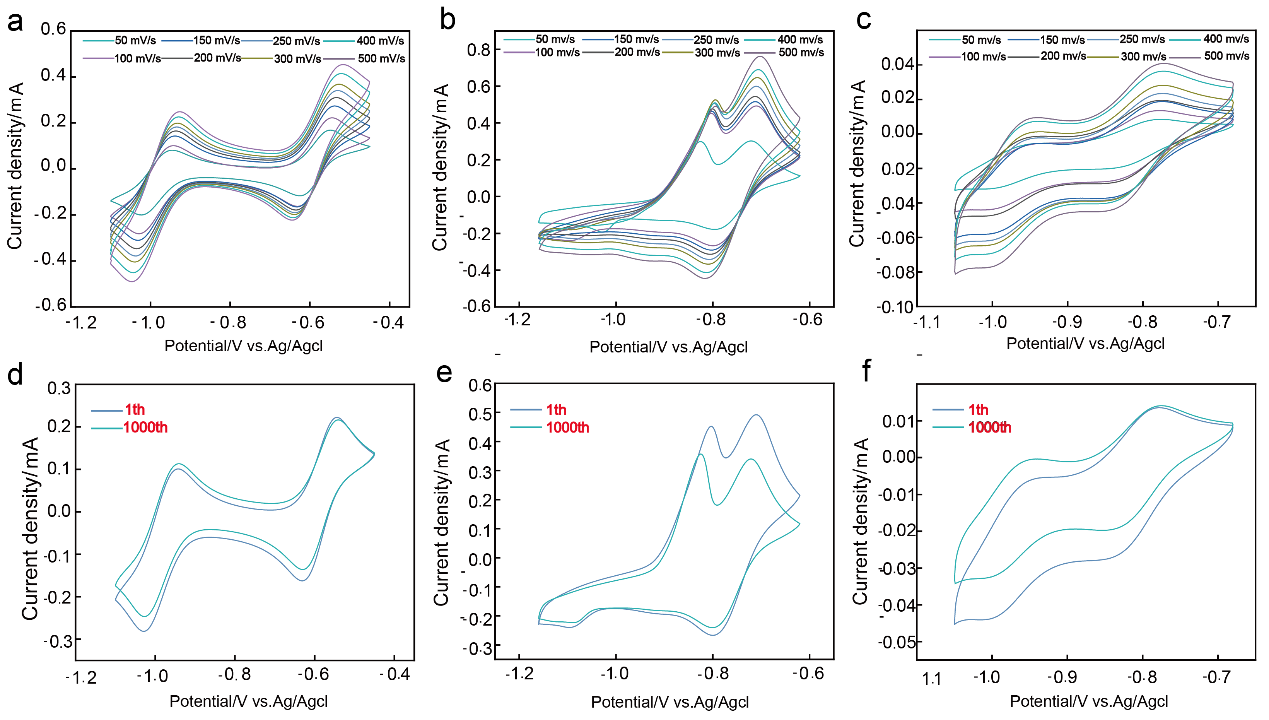


**Figure S9.** Electrochemical properties of viologen derivatives. Cyclic voltammograms (CVs) of 0.1 mmol L^-1^ SV (a), STV (b), and ETV (c) in H_2_O at different scan rates; Long-term CVs of the deposited SV (d), STV (e), and ETV (f) film at a constant potential scan rate of 100 mV s-1.


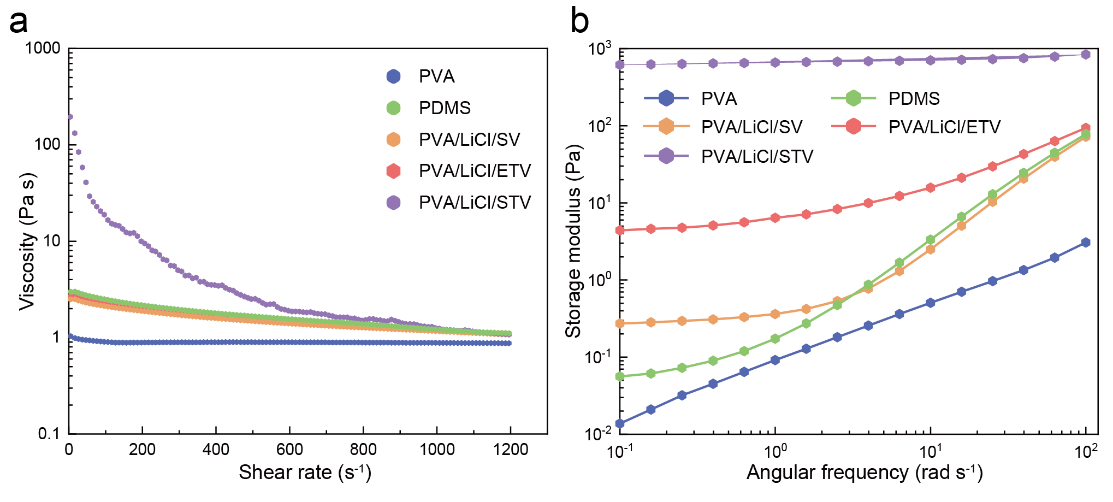


**Figure S10.** Rheological properties of multimaterial inks. a) Apparent viscosity as a function of shear rate for multimaterial inks. b) Shear storage modulus as a function of shear angle frequency.


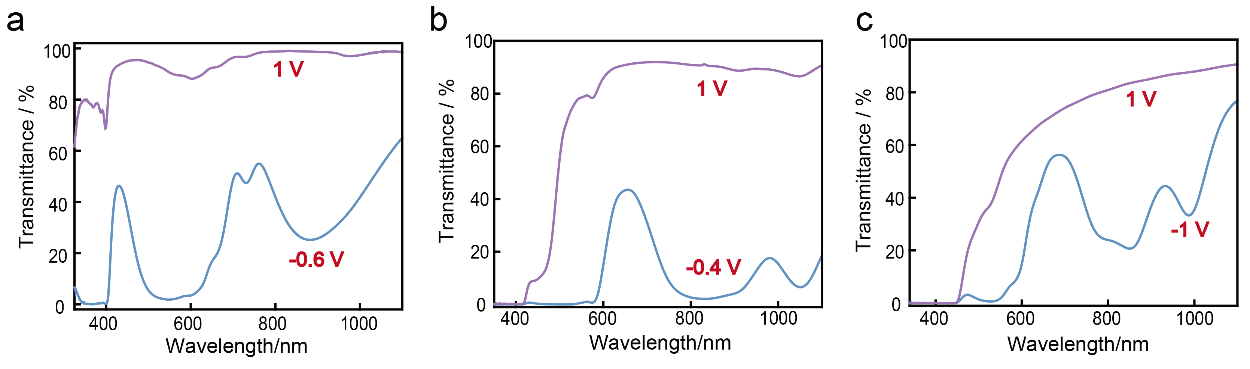


**Figure S11.** The transmittance spectra of FECDs based on SV (a), STV (b), and ETV (c).


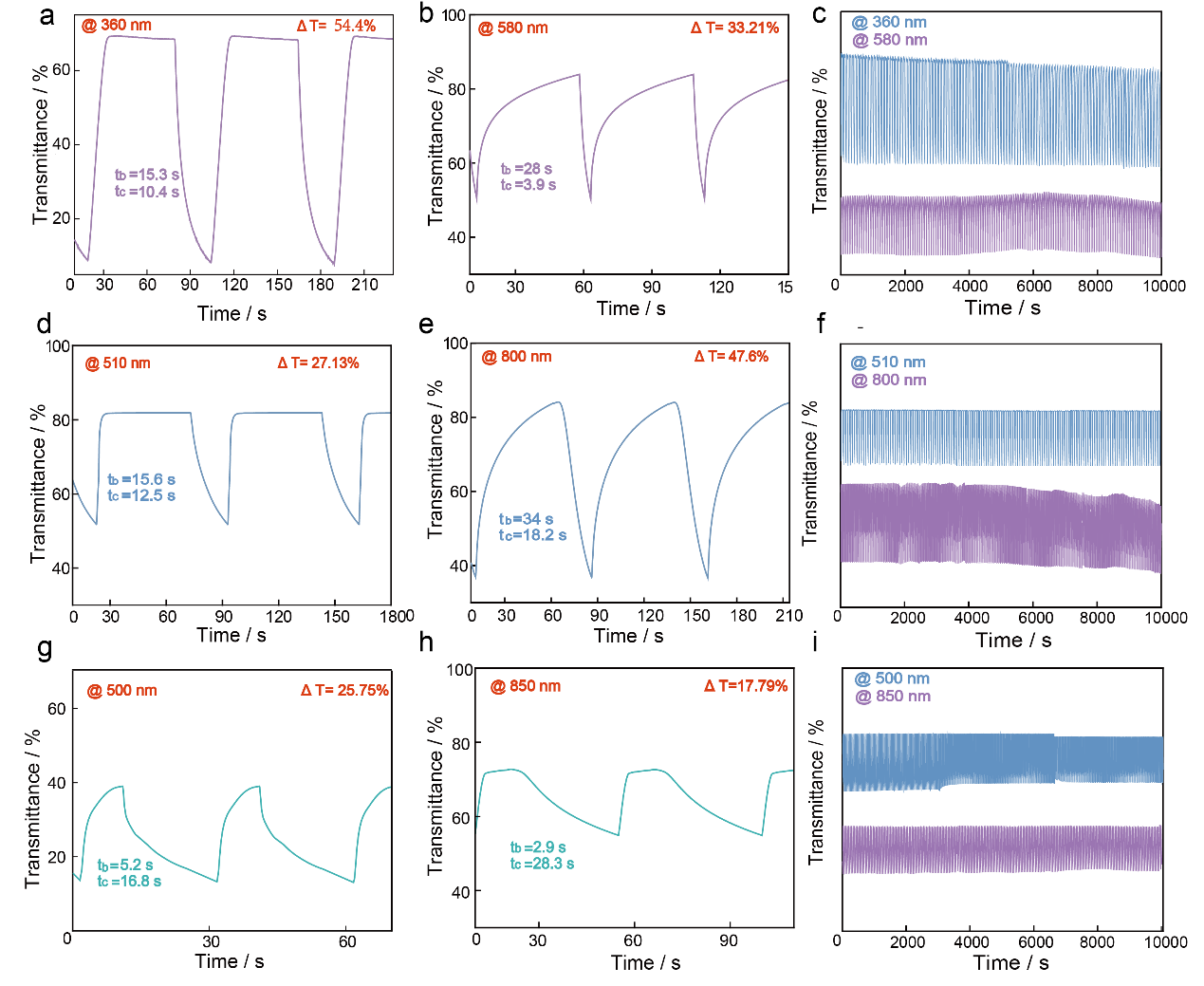


**Figure S12.** Electrochromic properties of integrated 3D printed hydrogels-based FECDs. Time-transmission curve of FECDs based on SV (a, b), STV (d, e), and ETV (g, h) at different wavelengths; Transmittance profiles of FECDs based on SV (c), STV (f), and ETV (i) against continuous long-term switching at different wavelengths.

**
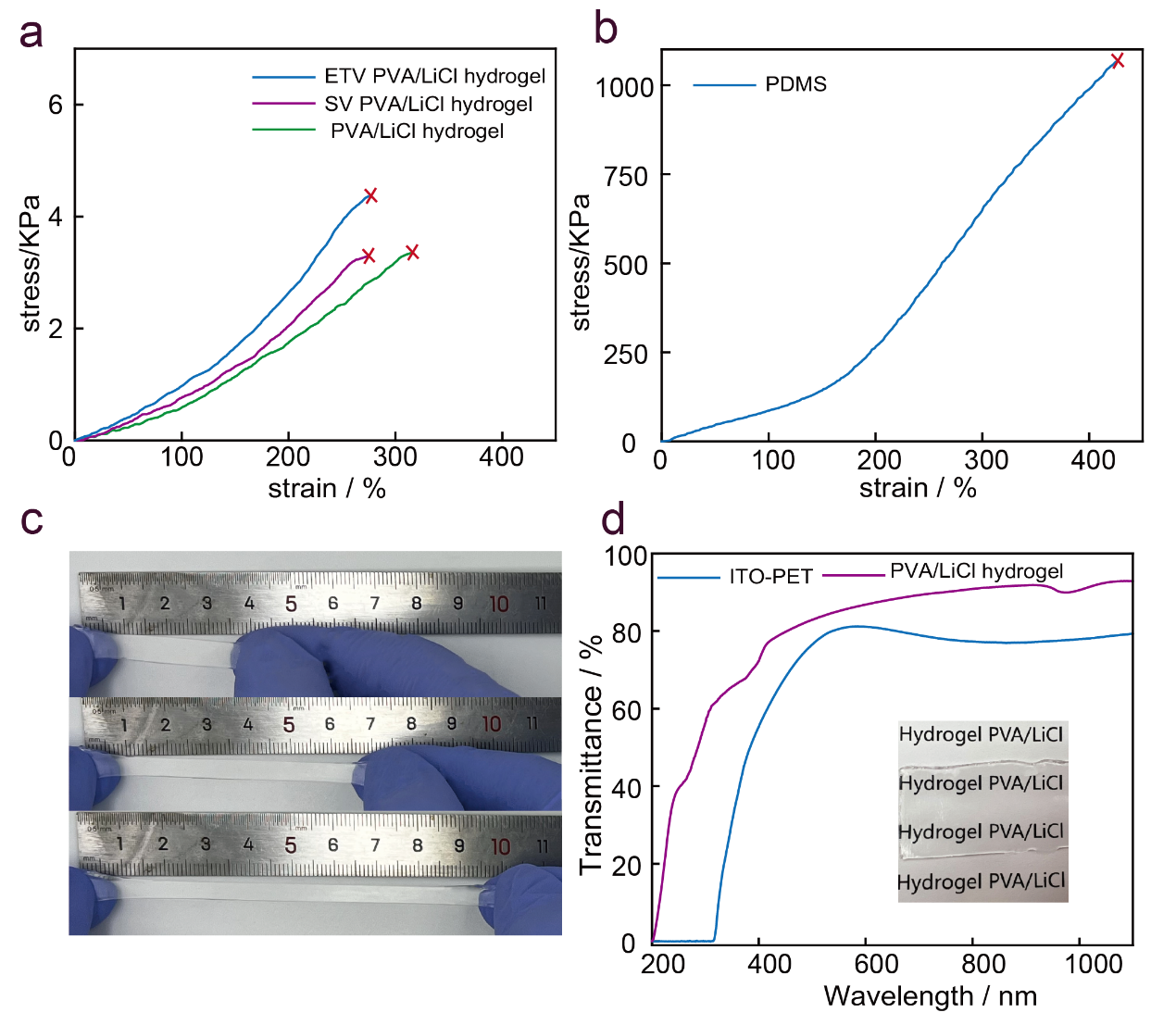
**

**Figure S13.**  a) Pressure-strain profiles of the SV, STV and PVA/LiCl hydrogel. b) Pressure-strain profiles of the PDMS. c) Photographs of a PVA hydrogel before and after stretching. d) Transmittance spectra of a blank PVA hydrogel and a piece of ITO-PET. The inset shows a photograph of a blank PVA hydrogel.

**References**

[1] C. DeBruler, B. Hu, J. Moss, J. Luo, T. Liu, A sulfonate functionalized viologen enabling neutral cation exchange, aqueous organic redox flow batteries toward renewable energy storage, ACS Energy Lett. 3 (2018) 663-668.

[2] M. Chang, D. Liang, F. Zhou, H. Xue, H. Zong, W. Chen, G. Zhou, Photochromic and electrochromic hydrogels based on ammonium- and sulfonate-functionalized thienoviologen derivatives, ACS Appl. Mater. Interfaces 14 (2022) 15448-15460.

[3] M. Kanagaraj, V. David, S. Vembu, K. Murugavel, H. Kuo-Chuan, Viologen-based electrochromic materials and devices, J. Mater. Chem. C 7 (2019) 4622-4637.
